# Socio-economic inequalities in suffering at the end of life among advanced cancer patients: results from the APPROACH study in five Asian countries

**DOI:** 10.1186/s12939-020-01274-5

**Published:** 2020-09-10

**Authors:** Chetna Malhotra, Anirudh Krishnan, Jing Rong Yong, Irene Teo, Semra Ozdemir, Xiao Hong Ning, Thushari Hapuarachchi, Gayatri Palat, Sushma Bhatnagar, Anjum Khan Joad, Pham Nguyen Tuong, Wynn Mon Ssu, Eric Finkelstein

**Affiliations:** 1 Lien Centre for Palliative Care, Duke-NUS Medical School, 8 College Road, Level 4, Singapore, 169857 Singapore; 2 Program in Health Services and Systems Research, Duke-NUS Medical School, Singapore, Singapore; 3 Division of Supportive and Palliative Care, National Cancer Centre Singapore, Singapore, Singapore; 4 Geriatric Department, Peking Union Medical College Hospital, Beijing, China; 5 National Cancer Institute Maharagama, Maharagama, Sri Lanka; 6 Department of Palliative Medicine, MNJ Institute of Oncology and Regional Cancer Center, Hyderabad, India; 7 Unit of Anesthesiology, Pain and Palliative Care, All India Institute of Medical Sciences, Delhi, India; 8 Department of Anesthesiology and Palliative Medicine, Bhagwan Mahaveer Cancer Hospital and Research Centre, Jaipur, India; 9 Oncology Center, Hue Central Hospital, 16 Le Loi, Hue City, Hue, Vietnam; 10 Clinical Research Division, Yangon General Hospital, Yangon, Myanmar

**Keywords:** Metastasis, Economic status, Inequalities, End of life

## Abstract

**Background:**

A systematic understanding of socio-economic inequalities in end-of-life (EOL) suffering among advanced cancer patients is required to inform efforts to reduce these inequalities as part of Universal Health Coverage goals.

**Aims:**

To assess inequalities in multiple domains of EOL suffering among advanced cancer patients – physical, functional, psychological, social, and spiritual –, using two socio-economic status (SES) indicators, education and perceived economic status of the household.

**Methods:**

We used cross-sectional data from surveys of stage IV cancer patients (*n* = 1378) from seven hospitals across five countries (China, Sri Lanka, India, Vietnam and Myanmar). We conducted separate multivariable linear regression models for each EOL suffering domain. We also tested interactions between the two SES indicators and between each SES indicator and patient age.

**Results:**

Patients living in low economic status households /with fewer years of education reported greater suffering in several domains. We also found significant interaction effects between economic status of the household and years of education for all EOL suffering outcomes. Age significantly moderated the association between economic status of the household and social suffering and between years of education and psychological, social, and spiritual suffering (*p* < 0.05 for all).

**Conclusion:**

Results highlight that SES inequalities in EOL suffering vary depending on the suffering domain, the SES indicator assessed, and by patient age. Greater palliative care resources for patients with low SES may help reduce these inequalities.

## Background

Of the 7 million cancer deaths each year, 5 million are from low and middle-income countries [1]. Without access to palliative care, these patients are likely to experience considerable end-of-life (EOL) suffering [2, 3]. The estimated number of cancer patients experiencing such suffering is expected to increase fivefold over the next four decades in low- and middle-income countries [4]. The Lancet Commission on Palliative Care and Pain Relief and the 2014 World Health Assembly resolution emphasized the urgency to reduce EOL suffering as part of Universal Health Coverage goal [5, 6]. A key tenet for achieving this goal is to reduce socio-economic status (SES) inequalities in EOL suffering. To do this first entails a systematic understanding of SES inequalities in EOL suffering.

Although considerable evidence exists regarding the presence of SES differences in health status and health care utilization globally [7], the evidence regarding SES inequalities within the context of EOL suffering remains fragmentary. Previous studies have reported that low SES cancer patients are more likely to experience higher mortality [8–13], depression [14] and other comorbidities [12, 13], and greater symptom burden [15] compared to higher SES patients. Low SES cancer patients are also less likely to receive palliative care [16, 17], and to die at home [17].

Not unique to any country, the SES inequalities in health outcomes observed can be attributed to various possible pathways. Firstly, low SES patients tend to have less access to healthcare resources [18, 19]. Even among countries with universal health coverage, SES inequalities continue to persist as patients from low SES are less likely to receive specialty care compared to high SES patients [20, 21]. Secondly, low education and health literacy levels may limit access to healthcare information among low SES patients [18]. This in turn affects their ability to navigate the healthcare system [13] and make informed choices regarding healthcare treatment and preventions [21]. Thirdly, low SES patients are likely to have weak social networks and experience social isolation, and hence can experience difficulty in obtaining useful medical guidance and support from their social ties [18, 22, 23]. Lastly, low SES patients tend to experience a wide range of stressors such as unstable employment, financial strain and low control at work; which can affect their health negatively through physiological mechanisms [20, 21]. However, within the context of multiple domains of EOL suffering – physical, functional, psychological, social, and spiritual –, a comprehensive understanding of SES inequalities is missing.

In this paper, we use data from five low- and middle-income countries in Asia. Our study has three aims. Our *first aim* was to assess differences in multiple domains of EOL suffering (physical, functional, psychological, social, and spiritual) by patients’ SES. One difficulty in assessing this is that there is no single best way to capture the effect of SES in its entirety on EOL suffering outcomes. Prior studies have measured SES using different indicators. These indicators are not interchangeable [18] and may not necessarily represent the same causal process even if they may be affecting EOL suffering similarly. For instance, education as an indicator of SES is related to health literacy and knowledge. Better-educated patients, therefore, are likely to have greater access to information regarding their illness and its treatment, be able to navigate the health care system, and to negotiate their treatment plans with their health care providers [19–21]. On the other hand, economic status of the household as an indicator of SES represents material standards and resources available to the patient to access quality health and social care [22]. There is literature to suggest that perceived economic status predicts health and mortality outcomes more strongly than an absolute measure of household wealth [20, 23–25]. Accordingly we used education and perceived economic status of the household as two distinct SES indicators.

Our *second aim* was to assess the interaction effects between our two SES indicators – education and perceived economic status of the household. Although past studies have mostly focused on the independent associations of SES indicators with health outcomes, it is possible that patients who are disadvantaged in both SES indicators have worse health outcomes compared to those who are disadvantaged in one/neither SES indicator (‘double jeopardy hypothesis’) [26, 27]. If this were true in the context of EOL, then patients with low education and living in (what they report to be) low economic status households would have worse EOL suffering outcomes compared to patients with one or no disadvantage. Others have reported that the effect of having economic resources is greater in the presence of higher education [28]. This implies that patients with low education could experience similar levels of suffering irrespective of the economic status of their household. We tested these contrasting hypotheses in our paper.

The *third aim* of the study was to assess whether patient age modifies the association between each SES indicator and EOL suffering. Some authors suggest that SES differentials are relatively small in old age as age-associated frailty may make older cancer patients more vulnerable to EOL suffering irrespective of their SES (age-as-leveller hypothesis) [29–31]. In contrast, the fundamental cause theory posits that at any given age, resources related to SES – cognitive skills, finances, power, and social standing – can be used to the same degree to access treatments. This implies that SES differences in EOL suffering would not vary by patients’ age [30]. On the contrary, the cumulative advantage/disadvantage theory suggests that SES differentials in EOL suffering outcomes will increase with age due to accumulation of advantage/disadvantage over the life-course [30–32]. We test these competing hypotheses within the context of different EOL suffering domains.

We used data from advanced cancer patients seeking treatment at major public hospitals in five low and middle-income Asian countries. The public hospitals in these countries offer health care at lower cost than private hospitals to ensure equity in access and health status irrespective of patients’ ability to pay. Thus, existence of SES inequalities in EOL suffering among patients attending public hospitals indicates inequalities that can potentially be reduced by targeting EOL palliative care services at these hospitals towards low SES patients.

## Methods

### Survey setting and participants

The study is part of a multi-country cross-sectional survey of advanced cancer patients titled “Asian Patient Perspectives Regarding Oncology Awareness, Care and Health (APPROACH)”. The present study analyzed data from patients seeking treatment in 7 hospitals across 5 countries – China (Peking Union Medical College Hospital, Beijing), Sri Lanka (National Cancer Institute of Sri Lanka, Maharagama), India (MNJ Institute of Oncology & Regional Cancer Centre, Hyderabad; All-India Institute of Medical Sciences, New Delhi; and Bhagwan Mahaveer Cancer Hospital & Research Centre, Jaipur), Vietnam (Hue Central Hospital, Hue), and Myanmar (Yangon General Hospital, Yangon). These hospitals are among the major public hospitals treating cancer patients in each country.

Between November 2016 and August 2018, we recruited a convenience sample of approximately 200 inpatients and outpatients at medical oncology departments of each participating hospital. Details on number of patients approached to take part in the study, found to be eligible and recruited at each study site are in Appendix Fig. 3. A total of 1378 participants from seven hospitals – three in India and one each in China, Vietnam, Myanmar, and Sri Lanka – were included in the analysis. Eligible patients included those with a diagnosis of metastatic (Stage IV) solid malignancy, aged 21 years or older, aware of their cancer diagnosis, had received anti-cancer treatment, were citizens of the country in which the survey took place, and able to speak and understand the language of the questionnaire.

The study was approved by the Institutional Review Board of the National University of Singapore (protocol reference: B-15-319) as well as ethics committees of each participating hospital.

### Survey questionnaire

Following written consent, patients were administered a face-to-face survey questionnaire in the majority language of their country/region. The survey questionnaire contained measures of physical, psychological, social and spiritual outcomes of EOL suffering among patients. We used standardized translations for all validated scales in the survey when available from developers, or translated and back-translated these scales according to developers’ instructions. Remaining survey questionnaire items, which were originally developed in English, were translated by a specialised external vendor and further reviewed for equivalence and cultural appropriateness by bilingual study team members. Survey questionnaire was pilot tested with 10 participants at each site before commencing the main survey.

Main independent variables were patients’ years of education and perceived economic status of the household. Perceived economic status (hereafter “economic status” for brevity) of the household was measured with one item asking patients to classify their household as being poor, lower middle class, upper middle class or wealthy. The latter two categories were combined to represent “higher economic status households”, while the former two categories represented “lower economic status households” and “middle economic status households” respectively. The validity of similar self-reported SES measures has been established [33, 34].

Following were the EOL suffering domains assessed:

- *Physical and functional suffering*: We assessed physical and functional suffering (i.e. lack of physical and functional well-being) using the physical and functional well-being sub-scales of the Functional Assessment of Cancer Therapy – General (FACT-G version 4). Each subscale contained 7 items with responses ranging from 0 (“Not at all”) to 4 (“Very much”) which were summed to obtain total scores for each sub-scale [35].
- *Pain severity*: We assessed pain severity by asking patients to rate their current, worst, least, and average pain over the past 24 h on a 10-point numeric rating scale ranging from 0 (“No pain”) to 10 (“Pain as bad as you can imagine”). The mean of these four pain scores was calculated as the pain severity score [36].
- *Psychological suffering*: We assessed psychological suffering using the 14-item Hospital Anxiety and Depression Scale [37, 38]. We did not assess psychological suffering in Myanmar and Vietnam.
- *Social suffering*: Social suffering (i.e. poor social well-being) was measured using the social well-being sub-scale of FACT-G. This subscale had 7 items each rated on a five-point scale ranging from 0 (“Not at all”) to 4 (“Very much”) [35].
- *Spiritual suffering*: We assessed spiritual suffering using the meaning/peace sub-scale (8 items) of the Functional Assessment of Chronic Illness Therapy – Spiritual (FACIT-Sp) well-being instrument. Again, each item on the scale was rated on a five-point scale ranging from 0 (“Not at all”) to 4 (“Very much”) [39]. We did not assess spiritual suffering in Myanmar.

Finally, we captured other patient demographics including their age and sex, and recruitment setting (inpatient/outpatient).

### Statistical analysis

We first described the distribution of patient socio-demographic characteristics and each outcome in the overall and country samples. We rescaled each outcome from 0 to 100 to generate an index score where 100 represented the highest level of suffering in each domain (i.e. lowest level of physical and functional well-being, highest pain severity, highest anxiety and depression, lowest social well-being, lowest spiritual well-being) and 0 represented the lowest level of suffering. We ran separate multivariable linear regressions for each EOL suffering domain with the main independent variables as perceived economic status of the household and years of education. All regressions controlled for patient age (mean-centred), sex, recruitment setting (inpatient/outpatient), and country (as dummy variables).

For each EOL suffering domain, we ran separate models assessing the interaction effect between patients’ economic status of the household and years of education. Then, in separate models, we tested the interaction between economic status of the household /years of education and patients’ age (< 60 years vs. > 60 years). All models incorporating interaction effects also controlled for patient age, sex, recruitment setting, and country (as dummy variables).

All analyses were conducted using Stata version 15.1.

## Results

Patients were an average of 53 years of age and 53% were female. The majority of patients were recruited in an inpatient setting. Patients had a median 9 years of education varying between 5 years in Myanmar and India and 12 years in China. Sample characteristics are presented in the aggregate and by country in Table 1.

**Table 1:** Sample characteristics

Characteristic	Full sample	China	India	Myanmar	Sri Lanka	Vietnam
	n (%)	n (%)	n (%)	n (%)	n (%)	n (%)
**N**	1378	183	601	194	200	200
**Age**, mean (SD), range	53.0 (13.1), 21–87	58.2 (13.5), 21–87	50.1 (12.6), 21–84	52.1 (13.2), 22–86	55.8 (12.8), 22–83	55.2 (11.1), 22–87
Missing	1 (0.1)	–	–	1 (0.5)	–	–
**Sex**						
Male	646 (46.9)	96 (52.5)	317 (52.8)	59 (30.4)	67 (33.5)	107 (53.5)
Female	731 (52.8)	86 (47.0)	284 (46.8)	135 (69.6)	133 (66.5)	93 (46.5)
Missing, if any	1 (0.1)	1 (0.5)	–	–	–	–
**Type of patient**						
Outpatient	461 (33.5)	59 (32.1)	339 (56.4)	34 (17.5)	15 (7.5)	14 (7.0)
Inpatient	915 (66.4)	122 (66.7)	262 (43.6)	160 (82.5)	185 (92.5)	186 (93.0)
Missing, if any	2 (0.1)	2 (1.1)	–	–	–	–
**Economic status of household**						
Low	405 (29.4)	16 (8.7)	204 (33.9)	65 (33.5)	55 (27.5)	65 (32.5)
Middle	642 (46.6)	75 (41.2)	288 (47.9)	84 (40.3)	114 (57.0)	81 (40.5)
High	308 (22.4)	90 (49.2)	106 (17.6)	27 (13.9)	31 (15.5)	54 (27.0)
Missing, if any	23 (1.7)	2 (1.1)	3 (0.5)	18 (9.3)	–	–
**Years of education**, median, IQR, range	9, 8, 0–25	12, 6, 0–20	5, 10, 0–25	5, 6, 0–15	11, 5, 0–23	10, 4, 0–20
Missing, if any	17 (1.4)	14 (7.6)	–	3 (1.5)		–

Table 2 shows summary statistics of each of the EOL suffering outcome measured and its variation by economic status of the household and education. Overall, in each domain patients from low economic status households and in the lowest education quintile reported greater suffering than those from high economic status households and in the highest education quintile. We saw the same trend among patient sample from each country, except in the case of India (for social suffering by economic status of household), China (for physical, functional and psychological suffering by education) and Vietnam (for physical suffering by education). Patients recruited from the hospital in China reported lowest suffering in each domain compared to patients recruited from hospitals in other countries.

**Table 2:** Distribution of end of life suffering outcomes in the sample and by socio-economic status

	Full sample	China	India	Myanmar†‡	Sri Lanka	Vietnam†
	mean (SD)	mean (SD)	mean (SD)	mean (SD)	mean (SD)	mean (SD)
**N**	1378	183	601	194	200	200
**Physical suffering** (*n* = 1321)	46.3 (22.6)	32.6 (21.6)	49.2 (20.8)	38.7 (20.0)	52.4 (25.4)	48.1 (21.0)
By economic status of household						
Low	50.9 (22.2)	45.5 (23.3)	48.2 (21.9)	44.1 (18.3)	65.8 (24.7)	52.6 (17.5)
Middle	46.4 (21.3)	33.6 (19.4)	50.3 (19.2)	36.6 (19.6)	47.8 (23.7)	49.7 (21.4)
High	40.0 (24.0)	29.8 (22.5)	47.5 (22.7)	34.8 (25.5)	45.5 (24.2)	40.4 (22.5)
By education						
Lowest education quintile	46.3 (21.6)	33.1 (21.4)	49.4 (20.2)	39.2 (19.0)	55.5 (25.7)	46.9 (19.8)
Highest education quintile	43.3 (23.2)	33.8 (27.1)	43.8 (21.6)	33.7 (23.6)	52.3 (25.9)	48.5 (20.9)
**Functional suffering** (*n* = 1322)	51.9 (22.2)	39.5 (22.0)	58.7 (21.7)	56.2 (17.7)	49.2 (19.0)	43.4 (20.8)
By economic status of household						
Low	58.9 (20.6)	49.8 (25.1)	62.8 (21.0)	60.7 (17.4)	60.3 (17.4)	46.8 (17.6)
Middle	50.9 (20.8)	42.5 (20.7)	55.4 (21.2)	55.5 (16.8)	46.9 (17.7)	45.1 (21.4)
High	45.2 (24.6)	35.2 (21.8)	59.7 (23.2)	46.0 (21.1)	37.9 (17.3)	36.8 (22.4)
By education						
Lowest education quintile	57.4 (20.5)	39.0 (21.2)	64.6 (17.2)	57.6 (17.6)	54.8 (16.6)	44.0 (21.4)
Highest education quintile	48.0 (22.5)	39.9 (21.2)	51.5 (22.7)	52.9 (21.6)	43.8 (18.2)	41.7 (23.5)
**Pain severity** (*n* = 1183)	28.9 (23.7)	15.1 (19.1)	33.2 (20.4)	25.3 (25.6)	34.7 (26.4)	30.2 (23.4)
By economic status of household						
Low	33.4 (22.8)	26.3 (19.5)	31.6 (20.7)	30.2 (25.3)	41.3 (26.3)	35.7 (21.4)
Middle	28.5 (23.7)	15.2 (19.3)	33.1 (19.1)	24.0 (27.0)	31.3 (26.6)	31.7 (23.5)
High	24.1 (23.7)	13.3 (18.5)	36.0 (22.3)	16.8 (23.7)	35.7 (24.7)	21.3 (23.4)
By education						
Lowest education quintile	29.4 (22.7)	15.7 (18.5)	33.3 (19.3)	25.8 (25.9)	31.1 (25.4)	33.7 (25.3)
Highest education quintile	26.2 (24.1)	13.4 (21.0)	31.5 (20.2)	18.0 (26.2)	28.7 (28.8)	29.6 (25.7)
**Psychological suffering** (*n* = 990)	36.3 (19.9)	19.5 (16.4)	40.5 (18.0)	–	39.0 (20.7)	–
By economic status of household						
Low	46.2 (21.1)	26.0 (17.6)	45.8 (20.0)	–	53.5 (22.1)	–
Middle	34.8 (17.7)	21.5 (17.1)	38.2 (16.3)		34.9 (17.7)	
High	27.6 (17.9)	17.0 (15.4)	36.0 (15.9)		28.7 (14.9)	
By education						
Lowest education quintile	37.8 (19.7)	18.2 (15.4)	41.1 (17.6)	–	44.9 (20.8)	–
Highest education quintile	33.5 (17.1)	22.9 (15.7)	35.3 (16.6)		36.3 (17.4)	
**Social suffering** (*n* = 1322)	31.6 (19.7)	23.1 (15.7)	38.4 (20.4)	33.7 (15.0)	27.0 (19.5)	22.8 (16.2)
By economic status of household						
Low	36.4 (19.6)	28.8 (13.4)	40.4 (20.3)	37.5 (16.5)	36.9 (20.3)	25.0 (16.6)
Middle	29.4 (19.1)	22.6 (16.1)	34.9 (19.8)	32.8 (14.2)	23.7 (17.8)	21.9 (17.6)
High	29.9 (19.9)	22.5 (15.7)	43.7 (20.5)	28.3 (14.2)	21.2 (18.3)	21.7 (13.1)
By education						
Lowest education quintile	34.9 (19.3)	23.6 (16.8)	40.7 (18.3)	34.2 (16.5)	32.1 (19.8)	22.1 (17.8)
Highest education quintile	30.9 (18.9)	27.5 (19.2)	35.0 (19.3)	34.3 (13.8)	24.1 (19.8)	20.3 (13.0)
**Spiritual suffering** (*n* = 1191)	36.8 (19.9)	25.0 (18.6)	39.3 (18.3)	–	43.6 (23.1)	33.3 (17.7)
By economic status of household						
Low	44.6 (20.3)	32.8 (22.5)	44.0 (17.5)	–	60.5 (23.4)	35.9 (17.7)
Middle	34.4 (18.3)	26.5 (17.5)	34.7 (17.5)		39.3 (17.8)	34.0 (17.8)
High	32.3 (20.1)	22.8 (18.4)	42.7 (19.0)		29.3 (17.4)	29.2 (17.1)
By education						
Lowest education quintile	38.8 (19.9)	23.9 (17.0)	40.5 (18.4)	–	52.9 (21.9)	35.9 (18.1)
Highest education quintile	32.8 (17.2)	29.0 (17.9)	34.9 (16.9)		30.8 (19.2)	29.6 (15.6)

Multivariable regressions showed that patients from low economic status households compared to high economic status households reported significantly greater physical (β = 6.94, SE = 1.82) and functional (β = 6.55, SE = 1.71) suffering, pain severity (β = 4.93, SE = 2.02), psychological suffering (β = 12.01, SE = 1.78), and spiritual suffering (β = 7.57, SE = 1.66) (*p* < 0.01 for all aforementioned outcomes). Similarly with increase in years of education, patients reported significantly less functional (β = − 0.61, SE = 0.12), social (β = − 0.44, SE = 0.11), and spiritual (β = − 0.26, SE = 0.12) suffering (*p* < 0.05; Table 3; our first aim).

**Table 3:** Association of socio-economic status with end of life suffering

VARIABLES	Physical suffering (*N* = 1282) Coeff (SE)	Functional suffering (*N* = 1282) Coeff (SE)	Pain severity (*N* = 1140) Coeff (SE)	Psychological suffering (*N* = 962) Coeff (SE)	Social suffering (*N* = 1282) Coeff (SE)	Spiritual suffering (*N* = 1162) Coeff (SE)
**Perceived economic status of household (Ref: High)**						
Low	6.94*** (1.82)	6.55*** (1.71)	4.93** (2.02)	12.01*** (1.78)	0.89 (1.54)	7.57*** (1.66)
Middle	3.04* (1.59)	1.49 (1.49)	1.30 (1.78)	2.09 (1.51)	−3.62*** (1.35)	−1.42 (1.44)
**Years of education**	−0.02 (0.13)	−0.61*** (0.12)	−0.09 (0.16)	− 0.04 (0.12)	−0.44*** (0.11)	− 0.26** (0.12)
**Sex (Ref: Males)**						
Females	4.73*** (1.27)	0.99 (1.19)	0.71 (1.44)	3.01** (1.20)	−1.56 (1.08)	0.72 (1.16)
**Age (mean-centred)**	−0.09* (0.05)	− 0.05 (0.05)	− 0.09 (0.16)	−0.05 (0.05)	− 0.09** (0.04)	0.02 (0.05)
**Type of patient (Ref: Outpatients)**						
Inpatients	−4.77*** (1.44)	7.83*** (1.36)	−2.16 (1.71)	−2.18* (1.29)	5.13*** (1.23)	1.61 (1.32)
**Country (Ref: China)**						
India	12.77*** (2.15)	15.97*** (2.01)	15.81*** (2.42)	17.28*** (1.81)	13.32*** (1.83)	11.53*** (1.89)
Myanmar	3.67 (2.68)	10.21*** (2.53)	8.51*** (2.65)	–	7.63*** (2.29)	–
Sri Lanka	18.28*** (2.33)	5.46** (2.19)	19.05*** (2.49)	17.43*** (1.94)	2.49 (1.98)	16.55*** (2.04)
Vietnam	15.08*** (2.30)	−0.33 (2.16)	14.62*** (2.47)	–	−2.72 (1.96)	5.81*** (2.02)

*** *p* < 0.01, ** *p* < 0.05, * *p* < 0.1

**Fig. 1:**
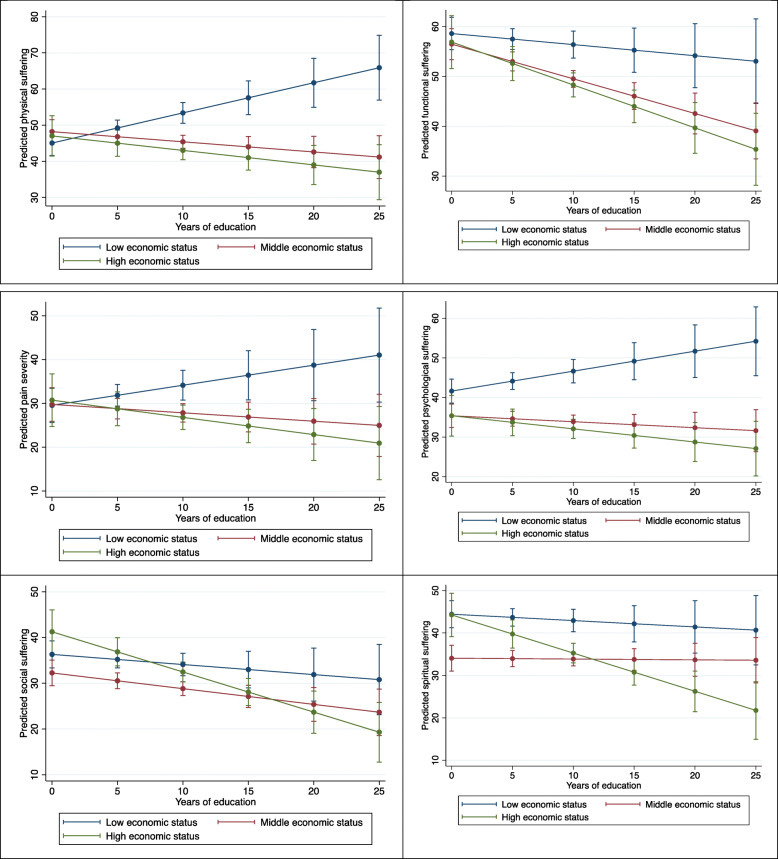
Interaction between economic status of the household and years of education

Interaction models showed significant interaction effects between economic status of the household and years of education for all EOL suffering outcomes (p < 0.05 for all; Fig. 1; our second aim). Full models are in Appendix Table 4. For the physical, functional and psychological suffering and pain severity outcomes, at more years of education, patients from low economic status households experienced greater suffering compared to those from middle and high economic status households. Conversely, patients with no education, irrespective of the economic status of their household, reported similar levels of suffering. Social and spiritual suffering did not vary significantly with education for patients from low economic status households, but reduced with more years of education for patients from high economic status households.

**Fig. 2:**
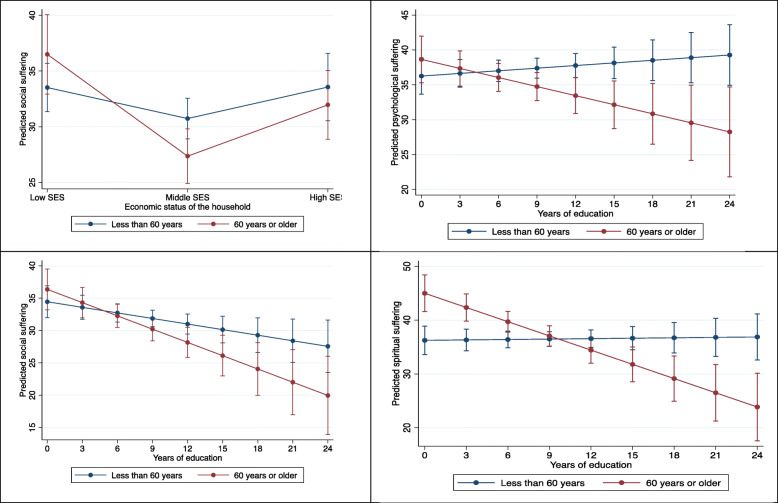
Moderating effect of age on the association between SES and EOL suffering

Age did not moderate the association of economic status of the household/years of education with physical and functional suffering and pain severity. Age however significantly moderated the association between economic status of the household and social suffering, and between years of education and psychological, social, and spiritual suffering. We found that older (> 60 years) patients from low economic status households reported greater social suffering than those from middle economic status households but younger (< 60 years) patients reported no difference in social suffering based on economic status of their households. Older patients with fewer years of education reported greater suffering in the social, psychological and spiritual suffering domains compared to those with more years of education but younger patients reported no such difference based on years of education (Fig. 2; full models in Appendix Tables 5 and 6; our third aim).

## Discussion

Our study extends existing literature on SES inequalities at EOL in several ways. First, we demonstrated the presence of SES inequalities across EOL suffering domains among patients attending public hospitals in five Asian countries, suggesting that we should strengthen EOL cancer services in these public hospitals to improve physical, functional, social and spiritual outcomes among low SES patients.

Second, we demonstrate not only the independent association of two SES indicators (education and economic status of household) with EOL suffering outcomes, but also the interaction effects between two SES indicators. Results did not find that patients with low education and low economic status households always had greater EOL suffering compared to others (double jeopardy hypothesis). We did find that patients with high education and living in high economic status households experienced a double advantage of the two SES indicators. Higher education coupled with sufficient financial resources may enable patients to access health and social care outside of the public hospital resulting in lower EOL suffering across all dimensions. On the other hand, without the benefit of education, patients may not fully be able to comprehend illness and treatment related information, may have poor adherence to medications such as for pain management and may not be able to participate in treatment related decisions [20, 21, 29, 40]. Financial resources therefore mattered little for these patients in the context of physical, functional, and psychological suffering and pain severity. Health care providers can therefore reduce such suffering among patients with low education by teaching them regarding their illness, ensuring compliance to treatment regimens and providing resources for psychological support.

We also found that SES differences in the more physical dimensions of suffering (physical and functional suffering and pain severity) remained constant with age thereby supporting the ‘fundamental cause’ theory [30]. It is likely that the use of resources such as money, knowledge and power - advantages conferred to patients with higher education and from living in high economic status households remained similar irrespective of their age [30, 41]. These resources are critical to accessing and navigating health care to reduce the physical dimensions of EOL suffering.

In terms of non-physical dimensions of suffering (e.g. social and spiritual suffering) at the EOL, results suggested that benefits of higher education are larger for patients from high economic status households than those from low economic status households. Previous studies have demonstrated that people with fewer financial resources have fewer social contacts because of lack of economic resources needed to engage with others [20, 42, 43]. This may result in greater social and spiritual suffering at the EOL. On the other hand, patients from high economic status households who may have developed more social networks [29] during their life course reported better social support (i.e. lowest social suffering) at their EOL. Effects were stronger when coupled with the positive effects of education on social networking and finding meaning/peace in life. Results also suggest that this disadvantage may accumulate over time resulting in low SES elderly experiencing greater suffering in non-physical dimensions. Low SES elderly may thus benefit from greater attention on these aspects by their health care providers.

The study has implications for health care providers. Hospital cancer services should conduct a holistic assessment of physical, functional, psychological, social, and spiritual suffering among patients, especially those from low economic status households and with lower education. Patients found to be experiencing suffering in one or more domains should be referred for targeted support from specialist services. Health care providers can also support patients with low levels of education routine consultations, by educating them about their illness and treatment option, and by monitoring their compliance to treatment regimens.

An important strength of our study is a large sample size drawn from countries from which little previous information was available regarding EOL outcomes. As we recruited patients from among those seeking treatment at major public hospitals in each country, our study samples are not representative of the target populations within the five countries.. For this reason, we are also unable to comment about the magnitude of SES inequalities between these countries. We are also unable to comment on differences in suffering scores between countries. For instance, lower suffering scores among patients from hospital in China compared to those from hospitals in other countries may be due to differences in hospital characteristics (e.g. availability of palliative care resources in the hospital) or due to characteristics of patients accessing the hospital. Cross-sectional data used for our analyses also limits causal inference in relation to the association between economic status of the household and EOL suffering. Our interpretations of results in this context are based on existing theories as well as driven by data and a reverse causation interpretation is plausible.

Our measure of economic status of the household was based on patient self-report. We did not use other widely used indicators of SES such as current income, past/present occupation or household wealth. Current income may be an inadequate proxy for contemporaneous financial resources available to patients to spend on their treatments. It is also likely that higher EOL suffering could lead to a decline in current income making a reverse causation hypothesis more plausible. In terms of occupation, there are difficulties assigning all patients to occupational categories due to differences in their meaning between countries. This would make an international comparison difficult. Measurement of household wealth is also challenging as information on spending patterns and household assets that can predict SES is lacking in many countries [44].

## Conclusion

Findings reveal SES inequalities in EOL suffering among advanced cancer patients in selected public hospitals in five low- and middle-income countries in Asia, with low SES patients dying ‘worse’ i.e. with more suffering, compared to patients from higher SES. Results further highlight that SES inequalities in EOL suffering vary depending on the suffering domain, the SES indicator assessed, and by patient age. Hospital cancer services in these countries can conduct a holistic assessment of these suffering outcomes and provide greater palliative care resources to patients from low economic status households and with lower education, especially elderly, to help reduce SES inequalities in suffering. Subsequent monitoring and evaluation of these EOL programs must also assess SES inequalities across multiple dimensions of EOL suffering taking into account the SES indicator being assessed and demographics of the patient population.

## Figures and Tables

**Fig. 3:**
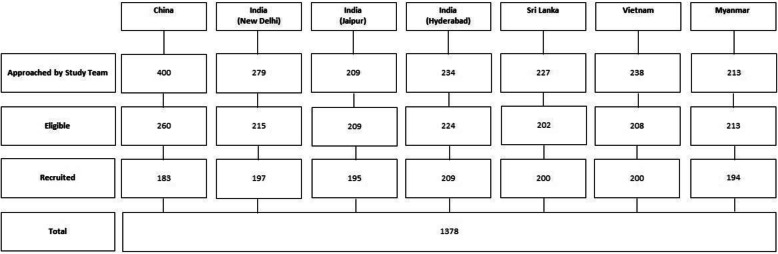
Flow diagram of response rates

## Data Availability

The datasets used and/or analysed during the current study are available from the corresponding author on reasonable request.

## Appendix

**Table 4:** Interaction models between economic status of the household and years of education

	Physical suffering (*N* = 1282) Coeff (SE)	Functional suffering (N = 1282) Coeff (SE)	Pain severity (N = 1140) Coeff (SE)	Psychological suffering (N = 962) Coeff (SE)	Social suffering (N = 1282) Coeff (SE)	Spiritual suffering (N = 1162) Coeff (SE)
**Perceived economic status of household (Ref: Low)**						
Middle	3.16 (2.30)	−2.13 (2.18)	0.21 (2.62)	−6.25*** (2.06)	−4.04** (1.97)	−10.38***(2.13)
High	1.98 (3.29)	−1.70 (3.11)	1.18 (3.54)	−6.24** (2.99)	4.95* (2.81)	−0.17 (3.02)
**Years of education**	0.83***(0.23)	−0.22 (0.22)	0.46*(0.28)	0.50**(0.22)	−0.22 (0.20)	−0.15 (0.21)
**Sex (Ref: Males)**						
Females	4.97***(1.26)	1.08 (1.20)	0.75 (1.44)	3.24***(1.21)	−1.61 (1.08)	0.66 (1.16)
**Age (mean-centred)**	−0.08 (0.05)	−0.05 (0.05)	− 0.07 (0.06)	−0.04 (0.05)	− 0.09**(0.04)	0.01 (0.05)
**Type of patient (Ref: Outpatients)**						
Inpatients	−4.15***(1.45)	8.14***(1.37)	−1.89 (1.72)	−1.66 (1.29)	5.39***(1.24)	1.91 (1.32)
**Country (Ref: China)**						
India	13.07***(2.13)	16.09***(2.02)	16.09***(2.42)	17.46***(1.81)	13.28***(1.82)	11.44***(1.89)
Myanmar	3.22 (2.67)	9.98***(2.53)	8.26***(2.65)		7.38***(2.29)	
Sri Lanka	17.85***(2.32)	5.20**(2.19)	18.72***(2.50)	16.99***(1.94)	2.11 (1.98)	16.06***(2.04)
Vietnam	13.98***(2.30)	−0.87 (2.18)	13.89***(2.48)		−3.18 (1.97)	5.38***(2.02)
**Perceived economic status of household X Years of education**						
Middle economic status of household X years of educations	−1.12***(0.27)	−0.47*(0.26)	−0.65**(0.32)	− 0.65***(0.25)	−0.12 (0.23)	0.13 (0.25)
High economic status of household X years of education	−1.23***(0.33)	−0.64**(0.31)	−0.85**(0.37)	− 0.84***(0.23)	−0.66**(0.28)	− 0.75**(0.30)

*** *p* < 0.01, ** *p* < 0.05, * *p* < 0.1

**Table 5:** Interaction model between economic status of the household and age

	Social suffering (N = 1282) Coeff (SE)
**Perceived economic status of household (Ref: Low)**	
Middle	−2.78*(1.44)
High	0.04 (1.93)
**Years of education**	−0.41***(0.11)
**Sex (Ref: Males)**	−1.35
Females	2.96**(1.20)
**Age 60 years or older**	2.98 (2.10)
**Type of patient (Ref: Outpatients)**	
Inpatients	5.09***(1.23)
**Country (Ref: China)**	
India	13.47***(1.83)
Myanmar	7.54***(2.29)
Sri Lanka	2.37 (1.98)
Vietnam	−2.74 (1.96)
**Perceived economic status of household X Age**	
Middle SES#Age 60+	−6.36**(2.60)
High SES #Age 60+	−4.58 (3.00)

**Table 6:** Interaction model between years of education and age

	Psychological suffering (*N* = 962) Coeff (SE)	Social suffering (*N* = 1282) Coeff (SE)	Spiritual suffering (*N* = 1162) Coeff (SE)
**Perceived economic status of household (Ref: Low)**			
Middle	−9.97***(1.38)	−4.67***(1.22)	−9.12***(1.32)
High	−11.82***(1.75)	−1.11 (1.54)	−7.55***(1.64)
**Years of education**	0.13 (0.13)	−0.29**(0.13)	0.03 (0.13)
**Sex (Ref: Males)**			
Females	2.97**(1.20)	−1.25 (1.08)	1.02 (1.15)
**Age 60 years or older**	2.39 (2.06)	1.92 (1.95)	8.75***(2.10)
**Type of patient (Ref: Outpatients)**			
Inpatients	−2.21*(1.28)	5.15***(1.23)	1.64 (1.31)
**Country (Ref: China)**			
India	16.80***(1.80)	13.53***(1.82)	11.41***(1.88)
Myanmar		7.58***(2.29)	
Sri Lanka	17.38***(1.93)	2.46 (1.98)	16.50***(2.03)
Vietnam		−2.88 (1.96)	5.62***(2.01)
**Years of education X Age 60+**	−0.56***(0.21)	−0.40**(0.20)	−0.91***(0.21)

*** *p* < 0.01, ** *p* < 0.05, * *p* < 0.1
